# Report of the Tuberculosis Commission1Presented July 8, 1909.

**Published:** 1909-08

**Authors:** John H. Pryor, Norman L. Burnham, William Gaertner, Charles R. Borzilleri


### Report of the Tuberculosis Commission.[1]

*To the President and Members of the Buffalo Academy of Medicine:*

Your commission on Tuberculosis respectfully reports as follows:

A recent investigation has shown that the increase in registration of those suffering from tuberculosis continues. From a tabulation kindly furnished by the Health Department a comparison is made for the first five months of each year for the last four years. In 1906, a total of 35 were reported; in 1907, the number was 138; in 1908, 230; in 1909, 263. Evidently many of the physicians in this city are endeavoring to comply with the request made by the Health Department.

An approximate estimate will reveal the fact that only about one-fourth of those fatally or severely afflicted are recorded. During 1908, the number of cases of tuberculosis reported and the number which became known by bacteriological examination was about 800, and there were 554 deaths certified as due to that scourge. The proportion of cases reported and the decided increase in the total number recorded is not as great as that exhibited in New York and other large cities where tuberculosis is regarded as a communicable and preventable disease. It is only too apparent that the Health Department does not obtain any information relative to the victims of tuberculosis in a large percentage of instances until death has occurred, and then no more is heard from that home until another unprotected unfortunate has succumbed to the malady.

Unless your commission has been misinformed no ordinance or law making the reporting of tuberculous cases compulsory has existed until the month of May, 1908. At that time two state laws providing for compulsory registration became effective. Neither of these statutes are yet operative or in process of enforcement in Buffalo. There are good reasons which may explain this remarkable delay. Hasty criticism would be largely inconsiderate or unfair. The State Department of Health did not issue the necessary literature, forms of instruction or blanks for requisite reports until many months after the Governor signed the rather comprehensive enactment. Furthermore, the local health department did not possess the additional funds actually required to comply with the laws.

Last winter when definite action was urged and attempted some of the provisions of the law were misunderstood and the difficulties of rendering it operative were rather ludicrously exaggerated. The corporation attorney was directed to prepare and send to the legislature a bill making Buffalo exempt from all its requirements. A member of your commission was afforded an opportunity to explain the law and answer objections and criticisms. Later it was decided not to transmit the proposed bill to

1. Presented July 8, 1909.

Albany and the necessity and propriety of the measures included in the statutes seemed to be recognised. The health department prepared an estimate of the probable cost of compliance and later the sum of $5 000 was appropriated by the common council for that purpose.

The amount was placed in the budget approved by the Mayor and will be available July 1, The literature containing cards of instruction and blanks for reports will be ready for distribution this week. The physician will be called upon to furnish information easily obtainable and its value will be of great importance from a statistical and sanitary standpoint. The health department will know the location of infectious foci and can learn their nature and extent. Effective methods in prevention can be introduced and scientific efforts to control the chief cause of death may be employed intelligently. We may in time learn something approximately accurate concerning prevalency and possibly a percentage of false death certificates may be obviated to use a mild and diplomatic expression. There may be also less pitiful excuse for evasion, lethargy and inaptitude. But the health department has endeavored to obtain certain essential agencies which if secured would make the receipt and collection of data of intense practical benefit. A clerk and two medical inspectors have been asked for to provide the inauguration of a system of investigation study and control. The work of medical inspectors will be imperatively necessary if a mass of statistics is to be used in a practical way.

Thus far no action has been possible because money to pay salaries has not been supplied. The common council evidently intended to create these positions but inasmuch as funds were not provided the Mayor cannot legally sanction any appointments until a proper form of procedure has been pursued. There seems to be no opposition to this increased expenditure for the improvement of the public health and it is expected that adequate funds will be allowed before July 1. How much delay will be caused in securing proper persons for the work only time will reveal. It now seems reasonably certain that the Health Department will be in a position to enforce state laws and begin a campaign against the greatest menace to the public health in the immediate future. Other powerful agencies recently created with a similar object will heartily cooperate in the vast struggle. If it is true that tuberculosis is preventable and unnecessary then the death rate in Buffalo is disgraceful and inactivity inexcusable.

The health department should be and can be the strong official leading factor in the modern crusade. It will have to assume new responsibility and will receive greatly increased power and additional facilities for effective work. Obviously your commission cannot offer any report on work not yet begun. Careful reading of the new laws leads to the surmise that enforcement may give rise to questions which may justify discussion after mature deliberation and the trial of experience. We urge the warm support of the profession and united complete fulfilment of more requirements in the interest of science and the welfare of the community. We recommend that a commission be continued to observe the movement along a new line of progress watch the results obtained, encourage professional support and interest and report to this body at the proper time. Finally, the members of the commission hold the opinion that any measure designed for the control and gradual eradication of tuberculosis will be only partially successful if increased provision for relief and care are not provided. Experience has demonstrated beyond dispute that prevention is proportionately effective to the extent that opportunity for proper care and isolation or seggregation is furnished. Increased hospital accommodation for the tuberculous is now the one imperative need in this city if the combat with a widespread communicable disease is expected to show results comparable with other cities where neglect and apathy have yielded to intelligent humane interest and persistent energetic attack.

Respectfully submitted,

John H. Pryor,
Norman L. Burnham,
William Gaertner,
Charles R. Borzilleri.

